# Stigma experienced by people living with HIV who are on methadone maintenance treatment and have symptoms of common mental disorders in Hanoi, Vietnam: a qualitative study

**DOI:** 10.1186/s12981-022-00491-y

**Published:** 2022-12-14

**Authors:** Ha V. Tran, Teresa R. Filipowicz, Kelsey R. Landrum, Ha T. T. Nong, Thuy T. T. Tran, Brian W. Pence, Vivian F. Go, Giang M. Le, Minh X. Nguyen, Ruth Verhey, Dixon Chibanda, Hien T. Ho, Bradley N. Gaynes

**Affiliations:** 1 The University of North Carolina-Vietnam Office, Hanoi, Vietnam; 2 Department of Epidemiology, University of North Carolina, Chapel Hill, USA; 3 Faculty of Environmental and Occupational Health, Hanoi University of Public Health, Hanoi, Vietnam; 4 Department of Health Behavior, Gillings School of Global Public Health, University of North Carolina, Chapel Hill, USA; 5 Center for Research and Training in HIV/AIDS, Hanoi Medical University, Hanoi, Vietnam; 6 Epidemiology Department, Hanoi Medical University, Hanoi, Vietnam; 7 Department of Psychiatry & Research Support Centre, University of Zimbabwe, Harare, Zimbabwe; 8 London School of Hygiene & Tropical Medicine, London, UK; 9 Faculty of Clinical Medicine, Hanoi University of Public Health, Hanoi, Vietnam; 10 Department of Psychiatry, University of North Carolina, Chapel Hill, USA

**Keywords:** HIV/AIDS, Stigma, Methadone maintenance treatment, Drug use, Common mental disorders

## Abstract

**Background:**

Stigma around human immunodeficiency virus (HIV), injection drug use (IDU), and mental health disorders can be co-occurring and have different impacts on the well-being of people living with HIV (PWH) who use drugs and have mental health disorders. This stigma can come from society, health professionals, and internalized stigma. A person who has more than one health condition can experience overlapping health-related stigma and levels of stigma which can prevent them from receiving necessary support and healthcare, serving to intensify their experience with stigma. This study investigates HIV, drug use, and mental health stigmas in three dimensions (social, internalized, and professional) around PWH on methadone maintenance treatment (MMT) who have common mental disorders (CMDs) including depression, anxiety, and stress-related disorders in Hanoi, Vietnam.Please check and confirm whether corresponding author's email id is correctly identified.The cooresponding author's email is correct

**Methods:**

We conducted semi-structured, in-depth interviews (IDIs) (n = 21) and two focus group discussions (FGDs) (n = 10) with PWH receiving MMT who have CMD symptoms, their family members, clinic health care providers, and clinic directors. We applied thematic analysis using NVIVO software version 12.0, with themes based on IDI and FGD guides and emergent themes from interview transcripts.

**Results:**

The study found evidence of different stigmas towards HIV, IDU, and CMDs from the community, family, health care providers, and participants themselves. Community and family members were physically and emotionally distant from patients due to societal stigma around illicit drug use and fears of acquiring HIV. Participants often conflated stigmas around drug use and HIV, referring to these stigmas interchangeably. The internalized stigma around having HIV and injecting drugs made PWH on MMT hesitant to seek support for CMDs. These stigmas compounded to negatively impact participants’ health.

**Conclusions:**

Strategies to reduce stigma affecting PWH on MMT should concurrently address stigmas around HIV, drug addiction, and mental health. Future studies could explore approaches to address internalized stigma to improve self-esteem, mental health, and capacities to cope with stigma for PWH on MMT.

*Trial registration:* NCT04790201, available at clinicaltrials.gov.

## Background

Human immunodeficiency virus (HIV) remains a burden in Vietnam, with an estimated 230,000 people living with HIV (PWH) in 2021 [1]. The HIV epidemic in Vietnam has been driven by injection drug use (IDU), and modeling suggests that an estimated 25% of new HIV infections in Vietnam occurred among male people who inject drugs (PWID) in 2017 [2]. Harm reduction programs including needle/syringe exchange programs, medication for opioid use disorder, HIV testing, and antiretroviral therapy (ART) initiation for PWID are recommended by the World Health Organization (WHO) to improve access to health care systems and mental health services [2] and are available in Vietnam. However, stigma against PWID remains a significant barrier preventing PWID from accessing HIV prevention and health care treatment [3, 4].

Substance use disorder (SUD), which is exemplified by worsened performance and harm to the people with this disorder and the entire community as well, has been reported as the most stigmatized condition [5], compared to some of the highly stigmatizing conditions (being homeless, being HIV positive, having a criminal record, alcohol use) [6], and people who have acquired HIV through injecting drugs face multiple types of stigma that affect their ability to access critical care for HIV and other diseases. PWH who inject drugs can be subjected not only to stigma related to drug use but also to the stigma around HIV infection [7]. Overall, all types of stigma detrimentally affect the mental health of the stigmatized person. Armoon and colleagues reported that people experiencing HIV-related stigma were more likely to be diagnosed with anxiety and depression than those who did not (1.89 times and 1.61 times, respectively) [8].

People experiencing multiple types of stigma may experience a greater impact of that stigma on their mental health. People who experience both HIV and drug use-related stigma experience greater rates of depression than people who experience only HIV-related stigma [9]. PWH who inject drugs can also experience mental disorders as the result of drug use or psychological distress related to living with HIV and drug use [10].

Studies in Vietnam on stigma around HIV and drug injection found that PWH and PWID both experienced stigma towards their HIV infection or drug use, respectively [11–13]. PWH who inject drugs suffered double stigma towards their health status and drug injection [14]. Levels of stigma towards people who abused drugs and are now on methadone maintenance treatment are highly associated with mental health disorders, unemployment, and HIV infection. Conversely, physical health problems, current drug use, and alcohol abuse were related to mental health problems among MMT patients [15]. While the stigma around HIV, IDU, or mental health distress was investigated separately with PWH and/or PWID, there haven’t been any studies on the stigma experienced by PWH who inject drugs and have mental health problems. This study aims to explore three types of health conditions (IDU, HIV, and CMD) among PWH who inject drugs and examine the combined effects of stigma on PWID with HIV and CMD.

This study is guided by literature around social stigma by Ahmedani [16], which describes three distinct dimensions of stigma: social, internalized, and professional stigma. *Social stigma* includes the attitudes and beliefs of the general public, family, and neighbors towards individuals with the stigmatized condition. The stigma around drug use is reinforced by moral attitudes toward drug use and the stereotyping of drug users as being bad [17]. *Internalized stigma* refers to the negative attitudes of an individual toward their health condition [18]. This type of stigma is correlated with emotions related to low self-respect and mental health challenges such as self-blame, embarrassment, depression, behavioral unmotivating, and social avoidance [19, 20]. Such negative thoughts significantly contribute to preventing stigmatized people from disclosing their health conditions and seeking needed help and support [21]. Professional stigma is a structural stigma that occurs when health care professionals stigmatize their patients with stigmatized conditions. The negative attitudes of health professionals toward patients’ HIV status and substance use disorders may contribute to suboptimal health care and outcomes for these patients [22].

Data for this analysis are part of the parent study, “Adaptation of the Friendship Bench counseling intervention to improve mental health and HIV care engagement outcomes among people living with HIV who inject drugs in Vietnam” [23]. This larger study adapts the Friendship counseling program and assesses the feasibility, acceptability, and fidelity of the culturally adapted intervention based on problem-solving therapy to improve mental health conditions among PWH on MMT who have CMD. To explore the most culturally appropriate way to adapt FB in Vietnam, one aspect of the study is understanding the current mental health of PWH on MMT, their coping strategies, and barriers to care. As a result, an understanding of the stigma around drug use, HIV, and mental health disorders is critical for the study team to address the stigma-related barriers for the FB administration in MMT clinics in Vietnam. The objective of this specific paper is to investigate different types of stigma around IDU, HIV, and CMD in three dimensions (social-, internalized-, and professional-stigma) among the study participants who were recruited from the parent study in 4 MMT clinics in Ha Noi, Vietnam in 2021.

## Methods

### Study settings and participant recruitment

This qualitative study was conducted from January to March 2021 in 4 MMT clinics in Hanoi, Vietnam. We conducted semi-structured, in-depth interviews (IDIs) with 12 PWH on MMT (hereunder-called patients), 5 of their family members, and 4 clinic directors (n = 21). We also conducted 2 focus group discussions (FGDs) with a total of 10 health care providers. Patients were 18 years or older, willing to participate in the study, and had a moderate severity score on the 21-item Depression, Anxiety, and Stress Scale (DASS-21) for at least one of the three CMDs (depression subscale score ≥ 14, anxiety subscale score ≥ 10, stress subscale score ≥ 19) [24]. Family members of the patients who were introduced to researchers by the patients and knew patients’ HIV and MMT statuses were eligible to participate in the study. Health care providers with at least 2 years of clinical experience with PWH and PWID in a clinical setting currently working at the 4 selected MMT clinics were eligible. Clinic directors at MMT clinics were invited for interviews.

### Data collection

Before interviews were conducted, participants provided written informed consent in Vietnamese. Each IDI and FGD lasted for a mean duration of 56 min and an hour 45 min respectively. Two interviewers with extensive experience working with PWH and PWID were trained in qualitative research methods to conduct all IDIs and FGDs in Vietnamese. All IDIs and FGDs occurred in a private place at one of the four study clinics or participants’ workplaces (for director interviews) and were audio-recorded. Audio recordings were de-identified, stored on a secure server, and transcribed verbatim with all identifying information removed to ensure confidentiality. Interviewers asked respondents questions about sources of stigma in general and about the stigma around having HIV, using drugs, and having common mental disorders. If participants affirmed that stigma existed, the interviewer probed about the respondents’ reactions to stigma and the ways they seek to access support and necessary healthcare services. Participants received monetary compensation for interviews. The study protocol, interview guides, and informed consent forms were approved by the Institutional Review Boards at the University of North Carolina and Hanoi Medical University.

### Data analysis

All transcripts were translated into English and analyzed in English. We conducted an applied thematic analysis using NVIVO 12.0 software [25]. Two investigators reviewed the transcripts to identify emergent themes for codebook development. The researchers then double-coded 20% of the transcripts to ensure intercoder reliability (ICR) was acceptable, reviewed and adjudicated discrepancies, and reached a consensus on coding decisions. The codebook was further refined before the final analysis through the addition of parent and child codes according to emergent themes. Thematic findings were discussed and agreed upon by the research team and are reported by thematic dominance. Thematic saturation is the point in data analysis when no new information emerges to address the research question [26]. In our study, thematic saturation was achieved within the study sample.

## Results

The study identified different types of stigmas experienced by PWH on MMT from different stakeholders’ perspectives including health care providers, family members, and PWH on MMT. These stigmas were categorized into three groups: social stigma, internalized stigma, and professional stigma.

### Social stigma toward HIV, drug addiction, and CMD

Most participants, including patients, family members, health care providers, and MMT directors, mentioned stigma towards HIV, drug use, and mental disorders from family and, more commonly, the community. Among most health care providers and MMT directors, stigma related to HIV and drug use were interchangeable. Participants often grouped these stigmas as if they were one stigma, or referred sequentially to one stigma and then the other. Three out of four MMT clinic directors and clinic staff members in one FGD provided their general opinion on community stigma toward their patients by mentioning stigma toward HIV and drug use at the same time. “In the community, there is a very clear stigma against people living with HIV and drug users” (Director ID 02, Female, 38 years old).

Particularly, since our study targeted PWH on MMT, the experience of stigma PWH who were on MMT shared was already the combination of stigma towards both HIV and drug addiction. For example, during one group discussion with health care providers, PWIDs might be subjected to social stigma toward HIV infection even though their HIV status were not known.

Now all information has been posted on the internet so they find anything there, but they can never stop stigmatizing. Many people ask if their parents have meals together with them or not, it proves that the stigma still exists. (FGD01).

The social stigma about drug use is still very strong. Four patients and one family member shared their specific experiences of stigmatization from the community with different forms (negative verbal language, attitudes, and unequal treatment) and severity levels (from avoidance and isolation to insults or breaks of confidentiality) towards to patient’s drug use history. These results demonstrate that much of the public continues to view drug use as a moral weakness, a sign of flawed character, or a danger to society. Even PWID attempting treatment with MMT is still stereotyped as addicts. “*Their attitude is still the old Vietnamese train of thought which sees drug addiction as a social evil. Although we take medication well, they think that we still use drugs, and say that we can never give up drugs … Because, for many years, I have been obsessed with hearing the word social evils, they consider me as an evil, the bottom of society*” [Patient DI 101, Male, 39 years old].

“When I go shopping, people don't want me to go into their stores, they ask me what I want to buy, who I want to meet. So, people unintentionally put a barrier in front of me, I don’t know if they want to keep a distance from me or if they stigmatize me. Normally, they don’t act like that to others. Maybe they’re afraid I will steal their things” (Patient ID 201, Male, 42 years old).

In addition to the social stigma towards PWIDs for their drug use and subjective stigmatization of HIV risk, a person with a drug use history was further stigmatized when his HIV status was exposed. Five patients experienced stigma from their families and relatives. Stigma was expressed via different gestures and verbal and non-verbal behaviors such as family members and relatives keeping a distance from patients. Three patients shared that they only had parents or wives emotionally and physically by their side when their HIV was exposed.

“I think it’s better to stay at home…In the past, when I had this disease, I lost everything… They still keep a distance. They don’t say it but through their gestures, I feel it… No, my parents don’t. Once my brother brought his child from Saigon to here to let my parents take care of his child, when I picked the baby up to cuddle her and played the game of lifting her in the air..then my brother called her “ [name], go play with your mom”, I knew right away that he didn’t want to let her play with me, I knew that they stigmatized me” (Patient ID 201, Male, 42 years old).

One patient shared his experience of stigmatized attitudes and behaviors from the community towards him. He felt that this stigma resulted from his neighborhood knowing he lives with HIV and a history of drug use. For one patient, his neighborhood demonstrated stigma by asking him to wear a mask to avoid transmission of HIV. Another patient experienced someone throwing his used cup of water.

“There was a guy who lived near my house, once I passed by his house, he called me in to drink water. At that time there were 5–7 people who were sitting there, and all used small cups, but he asked his wife to give me a big plastic cup and pour me water into that cup, then when I left, he threw the cup away.” (Patient ID 301, Male, 44 years old).

However, social stigma towards drug use and HIV has been reduced to a certain extent, especially when the community observes the positive change in PWIDs. Some participants’ family members noted that not everyone in the community expressed stigma toward HIV. Several study participants agreed that the levels of stigma towards HIV had been reduced significantly compared to the past. Two family members gave examples of social support for the HIV status of the patient in their families.

“Before, when I was still using drugs, I was stigmatized a lot, but now it is better. People see that I just stay at home to help my parents. When people pass by, they also nod heads and say hello to me and that also makes me happier” (Patient ID 201, Male, 42 years old).

The qualitative interviews provided evidence of the adverse effects of the community’s stigma of HIV and drug use on different aspects of a patient’s life. Nevertheless, stigmas around HIV and drug use are so often conflated that patients are unaware if they are being stigmatized because of having HIV or a history of drug use. One director and two patients shared the difficulty patients face in finding and keeping employment because of their drug use histories. Some patients refused to seek ARV treatment near their homes for fear of being discovered by their neighbors. In addition, patients were not the only ones to be stigmatized. Community members even stigmatized family members of PWIDs and hindered their social integration.

“…Generally, patients who are on both methadone and ARV treatment here, adhere to the treatment, and they are really afraid that people around them know about their status, their children’s emotions will be affected, and it will be difficult for them to find a job.” (FGD01).

Regarding common mental disorders, some patients discussed that there was a certain level of community stigma toward psychological distress among PWH who use drugs. People called PWH who use drugs with abnormal mental expressions with such stigmatizing words as “abnormal”, “lunatic”, “dump”, “insane”, “taciturn”, or “sluggish”.

“For example, when a depressed person passes by, people will say ‘A has just passed by, she looked taciturn’" (Patient ID 201, Male, 42 years old).

However, society's stigma towards CMDs was not as severe as it was for HIV and drug use. According to one patient, the community might view a person with a mental disorder as a threat but not stigmatize him/her the way they did to a person with HIV and/or substance use disorder.

“In my opinion, they [people in the community] think it’s better if that person [both on ARV, methadone and gets depressed] shouldn’t exist…… With psychological illness, people cannot self-control, so they might use a knife to kill someone when they have psychological morbidities, they cannot control their behavior… I think the level of precaution is the same, at a high level, but people don’t hate people with psychological morbidities as they hate people with HIV or drug users. (Patient ID 201, Male, 42 years old).

### Internalized stigma about drug use and HIV and CMD

This study found evidence of internalized stigma among the patients. Seven patients expressed thoughts of internalized stigma regarding their drug addiction and/or drug use. They perceived stigma but did not feel it necessary to understand whether they were being stigmatized for their HIV status or their drug use. The patients were vulnerable to directing stereotypes inwards. They were afraid of being stigmatized. It doesn’t even matter what type of stigma it is, but they fear being ostracized by the community. As such, they were aware of prejudices around drug use and or HIV carried by Vietnamese people and agreed with the negative stereotypes from which people developed negative feelings. Internalized stigma among patients was confirmed by the MMT director, health care providers, and family members.

“For example, I want to go to a cafeteria, and I go there, suddenly I feel that people stare at me. I have that feeling but indeed nobody looks at me like that. That is my self-stigma. I always have a feeling of guilty even if I am not guilty. It comes to me again and again because I still feel that people look at me with unsympathetic eyes and I am in a class that is despised by society. (Patient ID 203, Male, 56 years old).

A majority of study participants suggested that patients’ internalized stigma was caused by social stigma and the mistreatment they received because of their HIV status, history of substance use, and abnormal mental expressions as in the cases mentioned above. Some patients are also self-stigmatized for their negative changes in physical conditions, HIV, addiction, and personal reputation that their existence might create problems for their families.

“But now, I am scared to go anywhere, scared of people saying sort of that I am addicted. Therefore, I keep feeling nervous and anxious…. I am scared that when I become very sick, my body will look so horrible and I will bring a bad reputation to my family. Then my friends start to gossip and many more other things… I don't know because I just don't know how or what to do but anyways my lonely life makes me feel very depressed, in addition to that having no one to confide with is also really sad. Most of the time I think about the time when I get really sick, my body will look so horrible, and there is nowhere for me to rely on, I am alone…. First of all, I'm old. Second, I have this disease. The third is that I'm addicted. In general, I have experienced many things in my life, I also went to prison, then went to a rehab center, which means that my life has experienced many things that people cannot understand. Therefore, I hardly talk”. (Patient ID 102, Male, 35 years old).

All patients expressed a certain level of internalized stigma toward mental disorders by denying their mental health problems or stigmatizing mental disorders in general. They did not conceptualize their psychological symptoms as serious issues needing treatment. In some cases, patients’ internalized stigma was demonstrated by their avoidance of socialization, denial of seeking or receiving help, and self-devaluation. They reported that the shame associated with mental disorders, HIV/AIDS, and substance use makes them fear being noticed by people in the community.

“Normally for people with such issues, they don’t want to reveal that condition…In my opinion, people with mental issues seem to be more sensitive and they never admit that they are having trouble.” (FGD02).

“I don’t think I have a mental illness; I just think that is my nervous breakdown, but I don’t have a mental illness…. Mental illness is for mad people. Mental breakdown, people like me are mentally down, but cannot call us people with mental illness. (Patient ID 102, Male, 35 years old).

The combined impact of social and internalized stigma was perceived to intensify the development of psychological symptoms among PWIDs with CMDs. Seven patients shared their thoughts of depression, fear, anxiety, sadness/hopelessness, or lack of motivation, among other common psychological symptoms. These conditions might precipitate more serious problems, such as self-imposed isolation, hallucination, and self-harm. One patient shared his experience of suicidal ideation and intentions to harm himself.

“I think about this disease many times, and I feel scared of being stigmatized. If I am stigmatized by all the community, I can’t live anymore.” (Patient ID 401, Male, 51 years old).

“I feel sad and heavy like I couldn’t achieve the goal, I always think miscellaneous, then think that if they know I am like that or not so they will not accept me.” (Patient ID 302, Male, 44 years old).

“Sometimes I cried alone, even when I sit with you, I think about my depressed mind, think about myself, others, feel sad” (Patient ID 303, Male, 42 years old).

### Professional stigma towards HIV, drug addiction, and CMD

Stigma toward drug addiction was reported in health care providers in MMT centers who cared for patients. This stigma centered around substance use since substance users have long been stigmatized as being associated with criminal behaviors such as stealing, robbing, or violence due to cravings for substances or altered mental status due to intoxication. Healthcare workers did not openly express this stigma toward their clients, but they shared their concerns during the focus group discussion.

“They are not that bad but I and my family still watch out for them. We, the medical staff, kept talking well but actually, it was not good. If there is an addict in my neighborhood, for example, I surely will have to prepare several locks. Even when we say no discrimination, we never know when the addict is hyper, that is true it is.” (FGD01).

Health care providers shared that some health staff, including administrators and doctors, expressed their stigma towards HIV via unprofessional attitudes or behaviors such as raising their voices and showing negligence or contempt. These interactions were explained by the health staff’s lack of experience, training, understanding, or fear of their clients. However, such treatment might cause clients to lose their trust in the health staff and adversely affect the treatment process and clients’ adherence.

“However, at the reception desk, first, they sit behind the glass door, second, they are not familiar with patients. Even when receptionists see the patients’ pink health record book [HIV patients have a pink book which is different from that of other patients], some of them, I don’t say all, are trained inadequately so they may say unrespectful words to patients.” (FGD02).

Only one patient discussed negative attitudes from their clinician towards the patient’s situation, thoughts, and feelings. A family member also shared that, in some cases, medical staff did not meaningfully care for the HIV and MMT patients but only followed their consultation duties. One patient reported being hesitant to seek professional health care services.

“But if you let [name] and [name] lead us, we will punch in their faces right away, they don’t know anything, every time they open their mouths, they show disdain for us.” (Patient ID 401, Male, 51 years old). “I also heard patients telling their stories that sometimes the medical staff didn’t even look at their faces. They mean that the medical staff doesn’t care about patients, they only fulfill their responsibility.” (Family ID 1011, Female, 64 years old).

## Discussion

This study describes experiences of different stigma related to drug use and HIV from family, community, and health care providers towards PWH who are on MMT with a history of drug use and have CMDs. These stigmas were propagated by participants themselves, their family members, community members, and even health care providers towards their being with HIV, and history of drug use. Interestingly, HIV and drug use stigma are often used interchangeably and grouped, so much so that even the participants (PWH on MMT, family members, and health care providers) do not know what the root cause of their stigmatization—having HIV or using drugs. This may be, in part, because Vietnam and many other countries have a history of associating drug use with HIV [27]. As a result, it is difficult to separately analyze stigmas towards HIV or drug use. Participants with HIV and/or a history of drug use were afraid of being stigmatized and ostracized by their community, regardless of what specific aspect of their lives was being stigmatized. Among the three types of stigma discussed (HIV, substance use, and CMD), mental health-related stigma towards PWH on MMT is rarely recognized. Stigma toward mental health disorders is less intense and more peripheral, possibly because it is not perceived to be the “fault” of the PWH on MMT [28].

Although, there have been increased efforts to reduce stigma and discrimination toward substance use and HIV in Vietnam since 2000 in public and health care settings via media, education, intervention programs for HIV/AIDS prevention, and Drug Addiction Maintenance Treatment [29–32]. However, the stigma around substance use and HIV remain prevalent. Both society and family members have propagated stigma towards both PWH and PWID without distinguishing between the two types of stigma. The combined effect does not appear to be worse than one individually. Drug users are assumed PWH and PWH are assumed to use drugs. This study found that stigmas related to drug use and HIV are largely conflated, and the two types of stigma are inextricably linked. Findings of the impacts of social stigma on PWH who used drugs in our study are similar to others in that it reduces patients’ self-esteem, increases their social isolation, impairs their ability to find employment [11], increases mental health problems [33], hinders their efforts to access ART services [3, 12], and is related to their family also being rejected by the community. Additionally, this study found that social stigma is a strong factor in intensifying self-stigma, hindering patients from seeking social support to resume their normal lives.

The patients described and internalized negative stereotypes around HIV and SUD. Patients blamed themselves for contracting HIV and for their substance use that caused their families difficulties in their community. The consequences of internalized stigma around HIV and substance use caused some patients to isolate themselves from others, and not share their feelings and worries with others. These conditions consequently result in depression, anxiety, and stress. Indeed, existing literature documents that the application of stereotypes to oneself predicts a lack of self-respect and incapability of achieving personal goals [19]. This study’s findings reveal and support the impact of internalized stigma from another study in which participants experiencing high internalized stigma more frequently reported poor access to health care and support [34, 35]. Prior studies investigating stigma around PWID and PWH have been limited to describing the expression and consequences of stigma on well-being and levels of access to the health care system for stigmatized individuals [4, 7, 30, 33]. However, stigma reduction programs have not focused on addressing the internalized stigma of stigmatized subjects. Future research should invest in interventions for internalized stigma among PWID and PWH.

Previous research has found that stigma from health professionals can aggravate a patient’s self-stigmatization and can be an obstacle for patients seeking mental health counseling and care at health facilities [36]. The Vietnamese government and international programs have launched many innovative interventions to reduce the HIV stigma in health facilities [29, 37]. However, the manifestation of stigma from health professionals in ART and MMT settings is still documented in our study, as it is in others, and ranges from unwelcome attitudes to the patients, discrimination towards a patient’s medical diagnosis, lack of sympathy and understanding, treating the patients with unfriendly attitudes, staring at patients with disapproving expressions, fear, and distrust [13, 14]. We recommend fostering and expanding stigma reduction efforts in more burdened health care settings to achieve more efficient stigma reduction action.

Notably, the findings of this study demonstrated that stigma in the form of language surrounding mental health diagnoses and conditions intersects with the stigma around mental disorder statuses in a PWH with SUD. The stigma around mental health conditions can be easy to recognize in some contexts. However, in the context of substance use, mental health stigma may not be recognized as mental health stigma, which can be a stigma about abnormal behavior caused by substance use [30]. Therefore, the severity of mental disorders in patients with SUD and how it affects health outcomes may not be detected promptly. In this study, many patients’ internalized stigma was shown via their refusal to accept that they had mental disorders, avoidance of social interaction, and denial of seeking care for their mental health. They did experience internalized stigma expressed by their avoidance to contact with society, afraid that people will know about their mental health disorders. This represents an important intervention opportunity to address internalized stigma in this population.

Moreover, mental health services were not available in MMT clinics, therefore, patients go to MMT clinics for substance use treatment, but their mental health conditions were not diagnosed and treated in a timely or coordinated manner [38]. Even when patients were identified to have mental health disorders by MMT physicians, MMT clinics still referred them to the psychiatric department at the hospital. The referral could make patients feel embarrassed and stigmatized if they were diagnosed with a psychiatric disorder or made it harder for the patient to successfully approach mental health treatment [39]. We recommend that there should be mental health services, including screening, counseling, care, and treatment integrated into MMT clinics. This integration requires staffing health care providers in MMT clinics who are well-trained in common mental disorder diagnoses, care, and treatment.

There were three dimensions of stigma for all three health condition—HIV, IDU, and CMDs—that participants of this study may have had, which increased the impact of stigma on PWH on MMT. Future research should apply a quantitative approach to analyzing the interactions between and intersectionality of stigma around these three health conditions. This perspective will help develop strategies to improve the mental health of the PWH on MMT, build up their self-esteem and self-confidence, and empower them to cope with internal and external stigma.

Our study has some limitations. First, the study subjects were PWH on MMT and collected in four MMT clinics in Hanoi Vietnam, therefore findings may not be generalizable outside the context of a similar study population. However, data analysis of the paper reached thematic saturation, thus, was representative of PWH on MMT. Second, subjects with CMDs were screened with structured questionnaires by interviewers, not clinicians. This approach might create a certain level of bias around the psychological conditions of study participants, for example, because of their subjective descriptions of frequency and severity of symptoms. To mitigate this possible bias, the interviewers were thoroughly trained on how to perform the interview using the validated Vietnamese version of DASS-21.

## Conclusions

This qualitative study demonstrated the existence of different stigmas around HIV, IDU, and CMDs in three dimensions (societal, internalized, and health professional stigma) toward PWH on MMT who have CMDs in 4 MMT clinics in Hanoi, Vietnam. Social stigmas towards HIV and IDU are conflated such that participants did not differentiate between stigmas around HIV and drug use. The combined weight of social and professional stigmas intensifies internalized stigma and mental disorders and acts as a barrier to patients seeking necessary social and health support. In addition, the stigma around mental disorders may cause many patients to be in denial about their conditions and not engage in the uptake of care and support, subsequently refusing to share their conditions or seek social and medical support. Future stigma reduction interventions should address the internalized stigma of combined health conditions coexisting in HIV-infected MMT patients with CMDs.

## Data Availability

The datasets used and/or analyzed during the current study are available from the corresponding author on reasonable request.

## Abbreviations

ART: Anti-retroviral therapy
CDC: The Center for Disease Control and Prevention
CMDs: Common mental disorders
DASS-21: Depression, Anxiety and Stress Scale 21 items
FB: Friendship bench
FGD: Focus group discussion
HIV: Human immunodeficiency virus
HMU: Hanoi Medical University
IDI: In-depth interview
IDU: Injection drug use
IRB: Institutional Review Board
MMT: Methadone maintenance treatment
PWH: People living with HIV
PWID: People who inject drugs
SUD: Substance use disorder
ICR: Intercoder reliability
WHO: World Health Organization
